# Practical Medicine

**Published:** 1879-12

**Authors:** 


### PRACTICAL MEDICINE.

ARTIFICIAL DIGESTION.—MM. Vulpian and Mourrut. (*Archiv. gén. de Méd.*, October, 1879, p. 490.)

In presenting to the *Acad. de Méd.* a paper by M. Mourrut, entitled, *Recherches sur les Digestions Artificielles*, M. Vulpian communicated a note on the action of the digestive ferments employed in the treatment of dyspepsia. Experiments proved that different samples of pepsine do not have the same degree of digestive power, and that the addition of alcohol to an acidified pepsine or of the natural gastric juice retards digestion. Hence the conclusion that wines and elixirs of pepsine should not be used. M. Vulpian also showed that diastase and pancreatine, mixed with natural or artificial gastric juice are far from exerting upon amylaceous matter so energetic an action as when mixed in pure water with these substances. This fact of the influence of acid media upon these ferments was known, but it remained to be seen, if after having been in contact with the gastric juice in the stomach they would recover all their intensity of digestive action on entering the duodenum. This is the question which M. Mourrut proposed to solve. After having obtained negative results on adding to these ferments a few drops of hydrochloric acid, M. Mourrut neutralized them exactly. The diastase then recovered its activity and rapidly saccharified the starch which it had not modified before. The pancreatine, on the contrary, did not recover its saccharifying properties. Experiments relative to the peptonising action of pancreatine were less clear; but point to a certain extent in the same direction. Other experiments led M. Mourrut to believe that if alcohol does not prevent the action of pepsine upon azotized substances, it retards it, and finally that alcohol also retards the digestive action of diastase and of pancreatine.

JABORANDI IN YELLOW FEVER.—Dr. Giralt. (*Cron. Med. Quirurg de la Habana, and la France Médicale*, Sept. 27, 1879.)

During a practice of twenty-five years, the author has remarked that nearly all the cases of yellow fever, which from the beginning were accompanied by abundant sweating, terminated favorably, even when they passed to the second stage. The ideal of therapeutics then was in this malady, to have a medicament capable of provoking in this pathologic stage an abundant and prolonged perspiration. Unfortunately this medicament was lacking when the author collected his earlier cases, and it is only recently that this vacancy in the materia medica has been filled by the discovery of jaborandi.

Dr. Giralt regards yellow fever as an infectious disease or as an intoxication in which it is necessary to drive out the deleterious principle by the eliminatory organs—sudoriparous glands, kidneys, etc. One of these means of elimination is interdicted, since the secretion and emission of urine are suspended, but the two others remain open and it is precisely here that jaborandi has a double action upon them by the production of transpiration and salivation.

Dr. Giralt prescribed jaborandi for the first two cases of yellow fever he had since the popularization of this remedy. Dr. Monteresi, his pupil, has also used it and their patients became convalescent on the fifth day.

Jaborandi should be given from the invasion of the fever and continued throughout the first period and a part of the second, in the dose of 1. gm. in the twenty-four hours.

